# Red Blood Cell Extracellular Vesicle-Based Drug Delivery: Challenges and Opportunities

**DOI:** 10.3389/fmed.2021.761362

**Published:** 2021-12-24

**Authors:** Wararat Chiangjong, Pukkavadee Netsirisawan, Suradej Hongeng, Somchai Chutipongtanate

**Affiliations:** ^1^Pediatric Translational Research Unit, Department of Pediatrics, Faculty of Medicine Ramathibodi Hospital, Mahidol University, Bangkok, Thailand; ^2^Division of Hematology and Oncology, Department of Pediatrics, Faculty of Medicine Ramathibodi Hospital, Mahidol University, Bangkok, Thailand; ^3^Department of Clinical Epidemiology and Biostatistics, Faculty of Medicine Ramathibodi Hospital, Mahidol University, Bangkok, Thailand; ^4^Chakri Naruebodindra Medical Institute, Faculty of Medicine Ramathibodi Hospital, Mahidol University, Bangkok, Thailand

**Keywords:** therapeutic drug delivery, cancer, RBCEVs, extracellular vesicles, exosome, microvesicles, clinical application

## Abstract

Recently, red blood cell-derived extracellular vesicles (RBCEVs) have attracted attention for clinical applications because of their safety and biocompatibility. RBCEVs can escape macrophages through the binding of CD47 to inhibitory receptor signal regulatory protein α. Furthermore, genetic materials such as siRNA, miRNA, mRNA, or single-stranded RNA can be encapsulated within RBCEVs and then released into target cells for precise treatment. However, their side effects, half-lives, target cell specificity, and limited large-scale production under good manufacturing practice remain challenging. In this review, we summarized the biogenesis and composition of RBCEVs, discussed the advantages and disadvantages of RBCEVs for drug delivery compared with synthetic nanovesicles and non-red blood cell-derived EVs, and provided perspectives for overcoming current limitations to the use of RBCEVs for clinical applications.

## Introduction

Extracellular vesicles (EVs) are cell-derived vesicles present in bodily fluids that play an essential role in intercellular communication between tumor cells and other cells within the tumor micro- and macroenvironment (1). These secreted membranous vesicles are currently separated into three main classes on the basis of their size and biogenesis as follows: (i) apoptotic bodies (800–5,000 nm in diameter) released by cells undergoing programmed cell death; (ii) microvesicles (MVs; 50–1,000 nm in diameter), which are large membranous vesicles produced *via* plasma membrane budding; and (iii) exosomes (40–100 nm in diameter), which are small vesicles originating from the endosomal compartment (2, 3). Most cell types have been found to naturally secrete EVs under normal, physiological, and pathological conditions because of the dynamics of the cell membrane (4). Moreover, the biological functions of EVs are based on their surface composition and cellular cargo, which typically consists of bioactive molecules such as nucleic acids, lipids, and proteins. These molecules are delivered to adjacent and distant cells (5), and they lead to alterations of recipient cell fate and function and consequently modulate the surrounding microenvironment. EVs mediate functions in both healthy and disease states, as they circulate mini-messages throughout the body. For instance, healthy non-senescence mesenchymal stem cells can release EVs to repair damaged tissues and improve the stemness of the premature senescence stem cells (6, 7). EVs released from cells of the disease state contain specific molecules that could serve as biomarkers, and may also function as the mediators/aggravators of pathophysiologic processes (8–11).

EVs can be isolated from various human cells, including cancer cells, fibroblasts, epithelial cells, endothelial cells, immune cells, platelets, and red blood cells (RBCs) (12). RBCs can pass through all types of vessels and squeeze into capillaries with smaller diameters than normal RBCs for oxygen transport and carbon dioxide exchange in cells in all tissues throughout the body. RBC-derived extracellular vesicles (RBCEVs) are generated in circulation *via* shedding of the plasma membrane caused by complement-mediated calcium influx, followed by vesicle shedding (13). RBCEVs participate in several biological processes, such as nitric oxide (NO) homeostasis, redox balance, immunomodulation, and coagulation (14). Because they are produced from human RBCs, which practically lack both mitochondrial and nuclear DNA, RBCEVs therefore have a lower risk of horizontal gene transfer. RBCs have been widely used for blood transfusion for several decades, highlighting the potential safety and biocompatibility of RBCEVs (15). This review focuses on RBCEVs as robust nanocarriers with potential utility in future strategies as drug delivery platforms for clinical applications.

## RBCEV Biogenesis and Production

Normal RBCs have a flexible biconcave shape with a diameter of 7.5–8.7 μm and thickness of 1.7–2.2 μm (16). Phospholipids, including phosphatidylcholine, phosphatidylethanolamine, sphingomyelin, and phosphatidylserine, comprise 60% of the RBC membrane. The remaining content consists of lipidic compartments composed of cholesterol and glycolipids, representing 30 and 10% of the membrane, respectively (17). Furthermore, the RBC membrane also contains various proteins, such as peripheral proteins (e.g., spectrins) and integral proteins (e.g., band 3, glycophorins). Additionally, RBC membrane proteins can be classified by function into three groups: cytoskeletal proteins (e.g., spectrin, actin, protein 4.1), integral structural proteins (e.g., band 3, glycophorins), and anchoring proteins (e.g., ankyrin, protein 4.2) (17). Although hemoglobins are the major cytosolic proteins of intact RBCs, the cytoplasmic fraction also contains several proteins that serve as anti-oxidant and metabolic enzymes (18, 19). These proteins can release adenosine triphosphate (ATP) and NO into the intracellular environment (20, 21). Furthermore, RBCs are also the major vesicle-secreting cells in blood circulation. During their 120-day lifespan, RBCs lose ~20% of their hemoglobin content and membrane integrity during vesiculation. The physiological aging of RBCs, especially during the second half of their lifespan, accelerates vesicle generation (22). Indeed, vesiculation is one of the most important mechanisms by which RBCs eliminate any hazardous substances accumulated throughout their lifespan and prevent their early clearance from blood circulation (23, 24).

RBC membrane vesiculation is a homeostatic process activated in response to impaired or dangerous signaling machinery (25). This specific mechanism of vesiculation is related to the physical distortion of the RBC membrane caused by changes of the phospholipid organization (21). RBC vesiculation can be induced by ATP depletion, calcium loading, lysophosphatidic acid exposure, membrane protein disruption under pH 5.4 or heating, and cross-linking with diamide, resulting in interactions among the disrupted membrane proteins/lipids and shedding of the RBC membrane to generate spectrin-depleted MVs (26–30). Other stimuli known to induce RBC vesiculation include oxidative injury, endotoxin, cytokines, complement, and high shear stress (31). During ATP depletion, the activity of plasma membrane Ca^2+^ pumps is decreased, leading to increased Ca^2+^ concentrations within RBCs (26). Because plasma membrane enzymes such as flippase, floppase, and scramblase must maintain membrane phospholipid asymmetry, RBC scramblase increases anionic phospholipid exposure on the external leaflet of the plasma membrane (i.e., phosphatidylserine) and then releases vesicles (32). Moreover, circulating RBCs can remove membrane attacking complex pore components from the plasma membrane in a process requiring Ca^2+^, calpain activation, and spectrin disruption *via* vesiculation, resulting in EV formation (33). This membrane vesiculation may occur slowly during erythrocyte aging, in the blood circulation of patients with hemolytic RBC disorders, and in stored RBCs obtained for blood transfusion (34–36). Meanwhile, RBC vesiculation may also occur in response to energy depletion and compressive force on the RBC membrane (30).

Several stimuli have been applied to reproducibly generate RBCEVs as drug carriers, although it remains unclear whether different types of stimuli may lead to various RBCEV properties. The inducing factors used to stimulate RBCs to produce RBCEVs are presented in Table 1.

**Table 1:** Factors that induce RBCEV production.

**Inducing factors**	**Mechanisms**	**EVs characteristics**	**References**
Chemical reagents - Calcium ionophore - Lysophosphatidic acid - Phorbol 12-myristate 13-acetate	Calcium channel and protein kinase C activation leads to PS exposure and MV formation	RBC morphology changes from a spherical shape to a stomatocyte-, echinocyte- or discocyte-like shape. Negative surface charges on EVs depend on number of PS moieties	(30, 32, 37, 38)
Oxidative stress - tert-Butyl hydroperoxide	Oxidative stress-induced decrease in the osmotic fragility of RBCs, Hb oxidation, and EV formation	RBCEVs express PS and cell-specific band 3 epitopes on their surface, as well as enzymes involved in redox homeostasis and the complement-inhibiting proteins CD55 and CD59	(39)
Long-term storage	ATP depletion leads to changes in membrane mechanical properties and metabolic depletion following disturbances of membrane/cytoskeleton interactions	Accumulation of oxidized proteins	(40, 41)

*PS, phosphatidylserine; MV, microvesicle; RBC, red blood cell; EV, extracellular vesicle; RBCEV, red blood cell-derived extracellular vesicle; Hb, hemoglobin*.

## RBCEV Composition

RBCEVs consist of lipid bilayer spheroids (buds) with a diameter of 100–200 nm, and they are enriched in phospholipids, proteins, cholesterol, lipid rafts, hemoglobin, and acetylcholinesterase (37, 42). The components of RBCEVs are derived from RBC; however, they are not identical. Compared with their parental cells, RBCEVs lack cytoskeletal-linked molecules and possess lower membrane protein content, but they retain residual hemoglobins and metabolic proteins that contribute to their various biologic effects (25). The composition of hemoglobins, including HbA1c, of these vesicles is similar to that of intact RBCs (43). Table 2 summarizes and compares the main components of RBC and RBCEVs.

**Table 2:** Comparison of the major components of RBCs and RBCEVs.

**Composition**	**RBCs**	**RBCEVs**	**References**
**Size**	5–7 μm	100–300 nm	(16, 37)
**Membrane**			
- Phospholipid bilayer	PC, PE, SM, PS	PS, PE, PA	(17, 31)
- Lipids	Cholesterol, glycolipids	DAG, cholesterol	(17, 44)
- Proteins	Spectrins, band 3, glycophorins	Band 3, glycophorins, complement receptors, GPI-anchored proteins	(17)
- Genetic materials	DNA	N/A	(39)
**Cytoplasm**			
- DNA	Lack both nuclear and mitochondrial DNA	N/A	(15, 45)
- miRNAs (high abundance)	miR-451, miR-144, miR-486	miR-125b-5p, miR-4454, miR-451a	(46, 47)
- Proteins or markers	Hb tetramer–dimer, PRX oxidation-reduction, NOS	Hb, synexin, sorcin	(18, 19, 48–50)

*PC, phosphatidylcholine; PE, phosphatidylethanolamine; SM, sphingomyelin; PS, phosphatidylserine; Hb, hemoglobin; PA, phosphatidic acid; DAG, diacylglycerol; GPI, glycophosphatidylinositol; N/A, data not available; NOS, nitric oxide synthase; PRX, peroxiredoxin*.

RBCEVs contain lipid rafts and Fas-associated proteins to facilitate the action of a Fas–FADD–caspase 8–caspase 3 complex during RBC aging and death (51). The stomatin-specific lipid rafts present on RBCEVs are enriched in glycophosphatidylinositol-anchored proteins, i.e., complement decay-accelerating factor (DAF or CD55), membrane attacking complex inhibitory protein (CD59) (52, 53). RBCEVs also express CD47 on their surfaces to inhibit phagocytosis through an interaction with the macrophage inhibitory receptor signal regulatory protein alpha (SIRPα), thus preventing RBCEV clearance *via* endogenous elimination (54). RBCEVs are also enriched in synexin and sorcin, two proteins associated with stomatin-specific lipid rafts, as well as diacylglycerol and cholesterol as membrane lipids (24, 44, 48).

Notably, the components of RBCEVs can be modified during RBC storage (47, 55, 56). Previous evidence illustrated that RBCEVs released from stored RBC units had increased surface CD47 expression and intravesicular miR-4454 and miR-451a levels over time (47, 56). Concerning the membrane lipids, RBCEVs released after 4 weeks of RBC storage had higher ceramide, dihydroceramide, lysophosphatidylinositol, and lysophosphatidylglycerol levels, lower phosphatidylinositol and phosphatidylglycerol levels, but relatively unchanged phosphatidylethanolamine and lysophosphatidylethanolamine levels (55).

## RBCEV Applications for Drug Delivery

**Figure 1:**
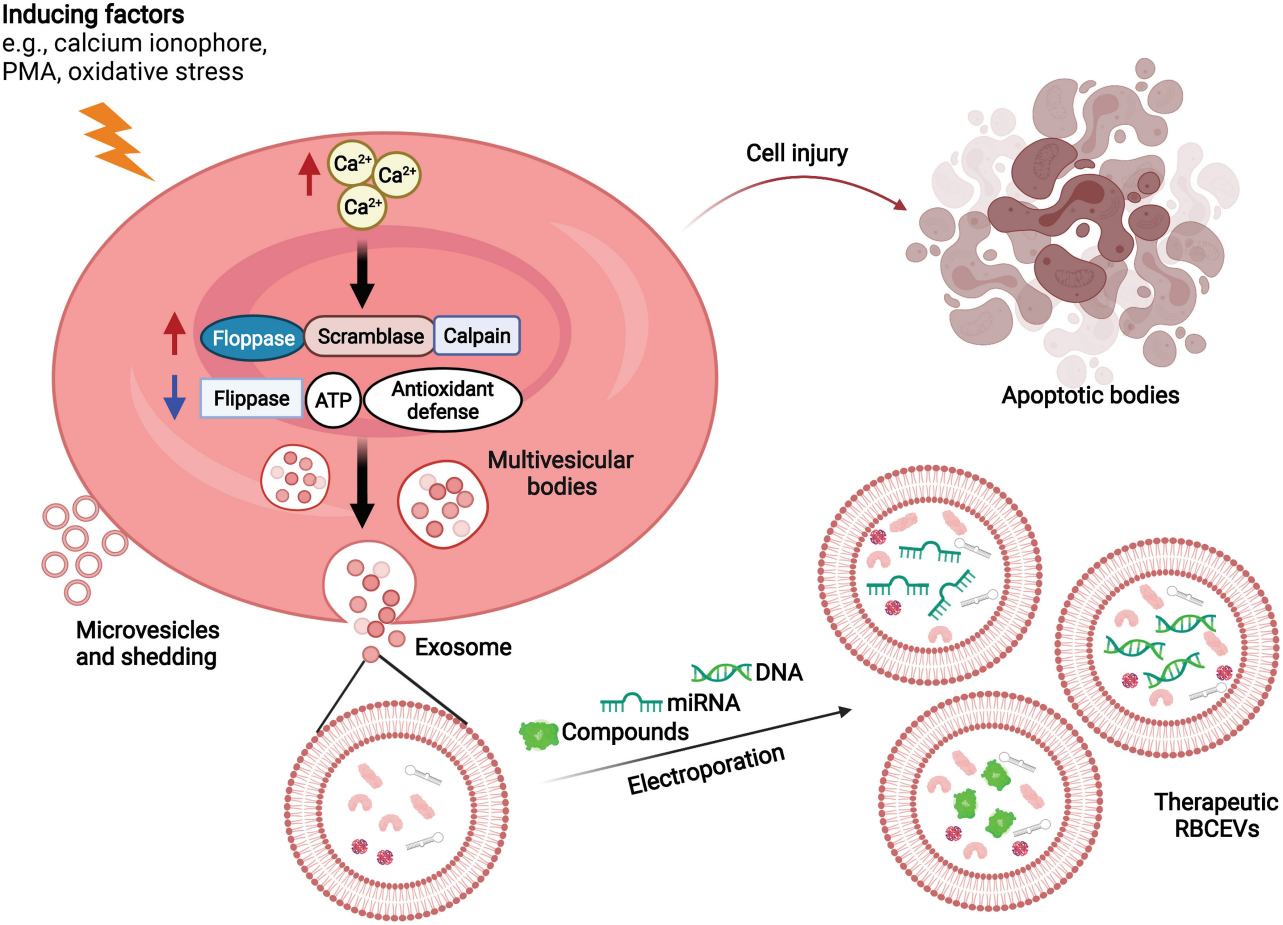
RBCEV production and cargo packaging for drug delivery. RBCs produce extracellular vesicles in response to increasing intracellular Ca^2+^ concentrations. Molecular therapeutic cargo (e.g., compounds, RNA, DNA) can be packaged into RBCEVs *via* electroporation for drug delivery. ATP, Adenosine triphosphate; PMA, Phorbol 12-myristate 13-acetate; RBCEVs, red blood cell-derived extracellular vesicles; RBCs, red blood cells.

Cumulative evidence suggests that RBCEVs can be applied in drug delivery systems (15, 57). The summary of RRBCEV production and cargo packaging for drug delivery is shown in Figure 1. RBCEVs have several advantages over conventional synthetic vehicles and non-RBC–derived EVs, all of which are discussed in this section.

### RBCEVs vs. Synthetic Nanovesicles

The desired properties of drug carriers include efficient cellular entry, near-natural physicochemical properties, and the ability to evade immune responses (58, 59). NVs are derived from natural and synthetic vesicular carriers. The types of natural lipid NVs include exosomes, virosomes, bacterial ghosts, and erythrocyte ghosts (60). Conversely, synthetic NVs were created to mimic the physicochemical properties of liposomes (61). Liposomes contain a lipid bilayer surrounding an aqueous core to allow the encapsulation and protection of hydrophilic molecules such as miRNA or DNA (62).

Liposomes have been widely used in drug delivery because their structure can effectively entrap various drugs and then transport cargo to target sites (63). This approach has demonstrated strong therapeutic efficacy in some cancer types (64, 65). However, liposomes have poor selectivity for cancer cells, resulting in severe systemic side effects (66). Conjugating liposomes with specific molecules, such as ligands, antibodies, or small molecules, improves selectivity and cellular targeting (67–69). By mimicking EV properties, synthetic NVs created from biomimetic phospholipid bilayers result in several improvements such as increased solubility, prolonged action, reduced toxicity, and lower adverse effects (66, 70–72). Nonetheless, the issues limiting the utility of synthetic NVs are immunorecognition as foreign substances and immune clearance by phagocytic cells (73).

In this regard, RBCEVs have proven extremely safe, and they can be used as robust carriers clinically because of their biocompatibility (74). Regarding biosafety, biocompatibility, efficiency, accessibility, and cost-effectiveness, RBCEVs are superior to conventional RNA delivery systems such as tripartite formulations with RNA, cationic polymers, and anionic liposome-encapsulated neutral lipopolyplexes (15, 75). Although conventional RNA delivery systems such as lipid nanoparticles are more stable than RBCEVs, they cause toxic side effects, and they are rapidly cleared from the circulation (76). RBCEVs have been used as carriers for RNA-based therapeutics to facilitate the effective delivery of both short RNA molecules and long mRNA molecules to their target sites for cancer therapy (47). RBCEVs loaded with RNA molecules display long-term stability and retain their functional capacity for long periods (77). Moreover, RBCEVs have great potential in drug delivery platforms because they can penetrate anatomical barriers and display sufficient binding (78, 79). This outstanding drug delivery platform carries special properties that make it suitable for drug delivery approaches (15). Further development of cancer-targeting peptide- or antibody-coated RBCEVs may result in improved target specificity and reduced adverse side effects in normal tissues. In addition to therapeutic agent delivery, RBCEVs can be applied to deliver ultra-small superparamagnetic iron oxide particles into human bone marrow mesenchymal stem cells for cellular magnetic resonance imaging to increase the performance of stem cell therapies (74). In addition, ^99m^Tc has been delivered to white blood cells *via* RBCEVs to observe organ inflammation in a mouse model using a gamma camera (80). This novel strategy using RBCEVs as delivery vehicles overcomes the limitations of traditional imaging including low intracellular labeling efficiency and biosafety concerns (74).

Production upscaling is perhaps easier for synthetic NVs. However, it should be noted that RBCEVs can be easily prepared at a relatively low cost from RBC units available in blood banks. Chemical induction (using modalities such as calcium ionophores) to enhance RBCEV release is an interesting scaling-up strategy for large-scale preparation and clinical applications (22, 81). Moreover, RBCEVs retain their stability and efficiency of delivery without any harmful effects even after multiple freeze–thaw cycles (82). As previously mentioned, CD47 expressed on the surface of RBCEVs prevents phagocytosis through an interaction with SIRPα (54), thus supporting the stability of RBCEVs after intravenous administration. In addition, EVs can efficiently penetrate the blood–brain barrier (83). Signaling molecules on the RBC membrane, which is a component of RBCEVs, can inhibit immune cell engulfment *via* an interaction between CD47 and SIRPα and defend against complement system attack *via* C8 binding protein, homologous restriction protein, DAF, membrane cofactor protein, complement receptor 1, and CD59 (84–86). This property of RBC membranes was applied to coat nanoparticles to increase their half-lives in blood circulation for drug delivery (87). Notably, it is feasible to prepare autologous RBCEVs for therapeutic agent loading (88).

Taken together, RBCEVs display advantages over conventional drug carriers in terms of high biocompatibility with limited immunogenicity, simple scaling-up, and high stability (15). Nonetheless, there are some disadvantages of RBCEVs for drug delivery. RBCEVs require a robust isolation method to separate them from blood cells and contaminating proteins. The heterogeneity of EV populations, including differences in EV size in the isolates, is nearly unavoidable depending on the isolation methods. A systematic comparison of EV isolation methods on the quality and quantity of plasma EVs indicated that ultracentrifugation (the gold standard) was the most appropriate method. Ultracentrifugation provided better EV purity compared to ExoQuick (System Biosciences), Total Exosome Isolation (TEI, Invitrogen), size exclusion chromatography (qEV), ultrafiltration, and exoEasy (Qiagen, membrane-based affinity binding) (89). However, the highest recovery rate was yielded by qEV (~60%), while ultracentrifugation and ultrafiltration yielded ~40% recovery rate (89). Polymer-based precipitation had impurity particles while exoEasy kit caused fusion and aggregation of EVs during the isolation process (89). Microfluidic and antibody selection platforms based on antigen-specific capture were successfully applied to isolate tumor-specific EVs (90). Unfortunately, microfluidic platform could separate EVs in a relatively small amount, i.e., 100 EVs per 1 μl and may cause EV aggregation during the isolation process (90). An optimized protocol for RBCEV preparation including an additional quality-control step is required to minimize batch effects and ensure the reproducibility of RBCEV applications. Notably, there is no study to clarify the normal range of EV concentration in the human body and it is unclear how various EV distributions in healthy or disease states might affect the efficacy of RBCEV-based therapy. For example, neuronal-enriched EV levels had not changed between healthy individuals and patients with Alzheimer's disease (91), so further study of RBCEV therapy in this disease context have no confounding from other EV distribution. Human lactoferrin could promote EV releasing from human adipose-derived stem cells (92), so the diseases with evidence of plasma lactoferrin changes might affect the interpretation of RBCEV therapeutic efficacy. The relationship between (exogenous) therapeutic RBCEVs and (endogenous) EV distribution should be clarified in future studies.

### RBCEVs vs. Non-RBC–derived EVs

Currently, which cell types represent the best sources of EVs for drug delivery remains unclear. Because EVs carry the membrane ligands and receptors of their parental cells, different cell types may produce EVs with differing delivery proficiency and targeting selectivity (81). RBCs, endothelial cells, monocytes, granulocytes, and platelets have been reported as cell sources for EV-based drug delivery (93). Conversely, fibroblast- and dendritic cell-derived EVs are not stably obtained from all subjects (94, 95), whereas cancer cell lines may release EVs that promote tumor development (96, 97). Various circulating cell type-derived EVs, especially those derived from nucleated cells, might contain genetic material, leading to horizontal gene transfer to recipient cells (98). Whole plasma is a major source of EVs that is easily obtained and readily available. However, whole plasma-derived EVs are heterogeneous, and they may contain several (unknown) substances (42).

Blood exosomes were engineered by co-embedding of drug and cholesterol-modified miR-21 inhibitor with high payloads into the lipid bilayer of exosomes (99). Moreover, superparamagnetic molecules and targeting proteins/peptides were loaded into the exosome membrane using ligand-receptor coupling and electrostatic interactions to enhance delivery to tumor cells and then inhibit tumor growth (99, 100). For example, in Parkinson's disease treatment, the engineering blood exosomes were applied to loaded dopamine by a saturated solution incubation method into blood transferrin receptor positive exosomes which were purified by multiple superparamagnetic nanoparticles labeled with transferrin (101, 102). Additionally, in type 2 diabetes mellitus treatment, a potential therapeutic peptide BAY55-9837 was loaded into exosome and coupled with superparamagnetic iron oxide nanoparticles with pancreas islet targeting activity to increase insulin secretion (103).

Interestingly, several properties of RBCEVs allow them to overcome the limitations of other cell source-derived EVs. First, RBCs are easily obtained and stored for prolonged periods after blood transfusion. Second, RBCEVs have long been present as a hidden component of transfused RBCs, which highlights their safety and biocompatibility. Third, RBCEVs have a low risk of horizontal gene transfer during delivery because RBCs lack nuclear and mitochondrial DNA (44). Finally, RBCEV release can be triggered by several processes, such as membrane complement activation and calcium influx. This EV release process can be applied to produce a large number of RBCEVs for experimental and clinical applications (81). Nonetheless, RBCEVs should be considered a blood product, and as such, blood group compatibility must be considered. In this regard, autologous RBCs can be an ideal source of EVs to avoid blood group incompatibility or immunorecognition.

For allogeneic treatments in patients with cancer, RBCEVs are safer than plasma EVs because cancer and immune cells generally release an extremely large number of cancer-promoting EVs into the circulation (96, 97).

A comparison of drug delivery characteristics between RBCEVs and non-RBC-derived EVs is presented in Table 3.

**Table 3:** Comparison of drug delivery systems between RBCEVs and other EVs.

**Property**	**RBCEVs**	**Other EVs**	**Refences**
Gene transfer	ND	Horizontal gene transfer	(15)
Drug content within EVs	ASOs = 200 pmol USPIO particles = 200 μg	Catalase = 0.1 mg/mL Curcumin = 2.9 g/g Paclitaxel = 5 μM	(15, 74, 104–106)
Number of EVs	1 × 10^11^	1 × 10^11^	(15, 106)
Packaging	Electroporation, hypoosmotic swelling	Electroporation, sonication, extrusion	(15, 74, 107)
Safety	Relatively safe	Oncogenic phenotypes	(15, 108)

*RBCEV, red blood cell-derived extracellular vesicle; EV, extracellular vesicle; ND, not detectable; ASO, anti-sense oligonucleotide; USPIO, ultra-small superparamagnetic iron oxide*.

### Drugs and Therapeutic Molecules Suitable for RBCEV-Mediated Transport

EVs, as natural carrier systems, efficiently deliver complex molecules including proteins, nucleic acids, lipids, and sugars similarly as their parental cells (109, 110). Moreover, because they have similar membrane properties as their parental cells, EVs can easily internalize into parental cells as well as target cells *via* clathrin-independent endocytosis and macropinocytosis (109). The different cell types may use different EV uptake pathways such as membrane fusion, phagocytosis, micropinocytosis, and endocytosis (111). EVs may bind to surface receptors of targeted cells, trigger intracellular signaling cascades, and then mediate EV uptake depending on EV composition and origin (112). For example, monocyte-derived dendritic cells could uptake the milk-derived EVs *via* dendritic cell-specific intercellular adhesion molecule-3-grabbing non-integrin (DC-SIGN) and mucin1 (MUC1) protein interaction and phagocytosis, but not the EVs derived from other sources or lacking MUC1 (113). Furthermore, EV uptake capability depends on the recipient cell types but not the donors (114). For instance, EV uptake of human colon carcinoma cells was mediated by clathrin-dependent endocytosis, but that in human lung carcinoma cells was mediated through neither clathrin- nor caveolin-dependent endocytosis (114). However, RBCEVs can internalize into cancer cells through their primary membrane components (i.e., phospholipids) (14, 115). To reduce the loss of drugs/molecules during transport to target cells, RBCEVs are designed to internalize drugs/molecules and reach the target cells without inducing immune system attack and drug/molecule loss, leading to increased treatment efficacy (116).

Drugs/small molecules are easily loaded into EVs. For example, the anti-inflammatory agent curcumin was loaded into exosomes *via* incubation at 22°C for 5 min (105). Curcumin-loaded exosomes were more stable than free curcumin *in vivo* following intranasal administration (105, 116). In addition, a heat shock technique for bacterial cell transfection (incubation on ice for 30 min followed by 42°C for 60 s) and five rounds of electroporation at 500 V using a 10-ms pulse were used to load miR-15a mimic/inhibitor into exosomes (117, 118). The EV loading protocol should be optimized to account for differences in properties among different EV sources (119). The size of EVs may also influence the size and number of loaded drugs/molecules (120). MVs can carry larger amounts of linear and plasmid DNA than EVs following electroporation (120). Additionally, smaller linear dsDNA (<750 bp) was loaded to EVs (85 ± 41 nm) at higher amounts than larger dsDNA (>1,000 bp) (120). Furthermore, miRNA loading into EVs has been optimized *via* incubation at 22°C for 2 h at pH 2.5 (121). Notably, unlike miRNA loading methods of RBCEVs that were comprehensively evaluated and optimized (15, 121), the protocols for small molecule drug-loading into RBCEVs require further studies in a systematic manner.

## Challenges and Limitations of RBCEV Applications

### Potential Side Effects

RBCEVs may have some cellular effects because they participate in several biological processes including oxidative stress, inflammation, NO homeostasis, thrombosis, and foam cell formation (122). In oxidative stress, RBCEVs can upregulate NADPH oxidase expression *via* the excessive production of ROS by activated neutrophils through respiratory burst (123, 124). This may change cytoskeletal and cell membrane asymmetry, leading to Oxi-ERY formation and hemolysis. This can ultimately cause cholesterol release, lipid peroxidation production, and protein and iron aggregation, thereby inducing vascular cell damage (24, 125). Furthermore, during inflammation, components on the membrane of RBCEVs, including cholesterol (induces inflammation reaction), iron and myeloperoxidase (catalyst and source of ROS production, respectively), hemoglobin (activates pro-inflammatory transcription factor), and phospholipase A_2_ (hydrolyzes phospholipid, resulting in inflammatory mediator production), may cause vascular inflammation, leading to coronary heart disease (21, 125–127). In NO homeostasis, RBCEVs can induce NO synthase, resulting in excessive NO production, enhanced ROS production, increased erythrocyte adhesion, and increased endothelial cell damage and dysfunction (128, 129). During thrombosis, RBCEVs have pro-coagulant activity, providing a site (i.e., phosphatidylserine) for prothrombinase assembly to accelerate the coagulant cascade from prothrombin to thrombin-mediated clot formation (130). When aged or damaged RBCs enter suicidal death (eryptosis), cell shrinkage and cell membrane blebbing and scrambling lead to phosphatidylserine (“eat me” marker) exposure on the outer cell surface and then induce macrophage engulfment, thereby stimulating foam cell formation (24, 131, 132). Additionally, cholesterol on the RBC membrane can trigger foam cell formation (133). However, these aforementioned causes of vascular damage may occur when blood vessels contain high numbers of RBCEVs (134).

Drug-loaded RBCEVs are designed to significantly reduce side effects on normal cells (135). For example, RBCEVs containing miR-125b anti-sense oligonucleotides effectively antagonized oncomiRs and suppressed tumorigenesis without any observable side effects in breast cancer (15). In addition, RBCEVs induce pro-coagulant activity *in vitro*, but the effect on thrombotic complications after blood transfusion is unknown (136).

### Specific Cellular Targets

In prior research, fluorescently labeled EVs could be up taken and accumulated by every cell type (137). However, EVs contain parts of the plasma membrane as their parental cells, including surface ligands and receptors. This fact highlights that EVs have specific interactions with target cells through several mechanisms such as direct fusion with the plasma membrane, endocytosis, binding to cell surface receptors and docking at the cell surface (111, 138, 139). For these mechanisms, interactions between the surface proteins of EVs and those of recipient cells, such as that between syncytin and its receptor major facilitator superfamily domain 2a, are required (140, 141).

RBCEVs bound to the target amino acid sequence can deliver drugs to specific cancer cells (135). Moreover, *Plasmodium falciparum*-infected RBC-derived RBCEVs loaded with drugs produced better therapeutic efficacy against malaria *in vitro* than normal RBCEVs loaded with drugs and free drugs (142). The specific interaction between EVs and cells depends on the origin of the EVs and the target cells under active processes (115).

### Half-Life and Shelf Life

The factors influencing RBCEV release include (i) RBC storage conditions (i.e., several weeks at 4°C in additive solutions), (ii) donor variability, and (iii) the leukoreduction method (89). First, during RBC period, ATP concentrations inside RBCs decrease, resulting in membrane skeleton destabilization and intracellular calcium increase and leading to vesiculation (143). Moreover, the loss of endogenous anti-oxidants during RBC storage causes a number of proteins to undergo oxidative degradation such as spectrin, beta-actin, glyceraldehyde-3-phosphate dehydrogenase, and band 4.1, leading to vesiculation processes (144, 145). Furthermore, oxidative modification has been observed in the hemoglobin-beta chain, which affects the function of hemoglobin (146). Similarly, storage at 4°C can inhibit the ATP-dependent activity of Na^+^/K^+^ cationic pumps, resulting in increased Na^+^ and Ca^2+^ concentrations inside cells and subsequently increased RBC vesiculation (147). Conversely, the size of RBCEVs changes during storage from 100 nm after 5 days up to 200 nm after 42 days (148). Second, donor-specific factors depend on the hematological profile, which affects the basal number of RBCEVs and level of hemolysis during packed RBC preparation. Third, the leukoreduction method affects the size and number of RBCEVs, as RBCEVs obtained *via* whole-blood filtration had a smaller diameter (<200 nm) and higher total count than those prepared using the buffy coat method (149).

The RBC half-life is 58 ± 1.5 days (150), whereas the clearance half-time of RBCEVs in peripheral circulation after injection using ^125^I-tagged RBCEVs is 44 min (83). In addition, RBCEVs remain stable and intact even after multiple freeze–thaw cycles, and they have long-term stability at −80°C without effects on the moiety, uptake, and genetic material loading capacity (135).

## Future Prospects and Conclusion Remarks

Because the applications of EVs are not obvious, the International Society for Extracellular Vesicles aimed to standardize and develop recommendations and guidelines to improve the reproducibility of EV research (151, 152). EVs can deliver small molecules, nucleic acids, proteins, and metal nanoparticles for therapy and diagnosis (153). In addition, miRNA inside EVs is more stable than free miRNA because it is shielded from potentially damaging agents (154). Although EV-based drug delivery systems have limitations including a lack of standard isolation and purification methods, limited drug-loading efficiency, and insufficient clinical-grade production, EVs have a number of advantages, such as limited immunogenicity and cytotoxicity, stability in circulation, and specific cell targeting (153). However, there is no systematic study to define a normal range of EV concentration in a human body and this could be an important research topic in future. Also, several common medications may also affect the number of EV distribution in the body, for example, indomethacin (a non-steroidal anti-inflammatory drug for pain controlling), glibenclamide (a blood sugar lowering drug for diabetes mellitus treatment), clopidogrel (an anti-platelet medication for preventing blood clots) can inhibit EV biogenesis and release (155). These medications are potential confounding factors in the clinical EV studies.

RBCEV-based drug delivery was examined in several disease models (156). RBCEVs can be used to deliver RNA molecules to cellular targets and then release the material into the recipient cells (156). RBCEVs have been used delivery vehicles for gene therapy to cancer cells (26). RBCEVs have several advanced such as no risk of horizontal gene transfer (lack of both mitochondrial and nuclear DNA), extraordinary biosafety and biocompatibility, easy storage and transportation, and easy production in a large-scale and cost-effective manner (157).

**Figure 2:**
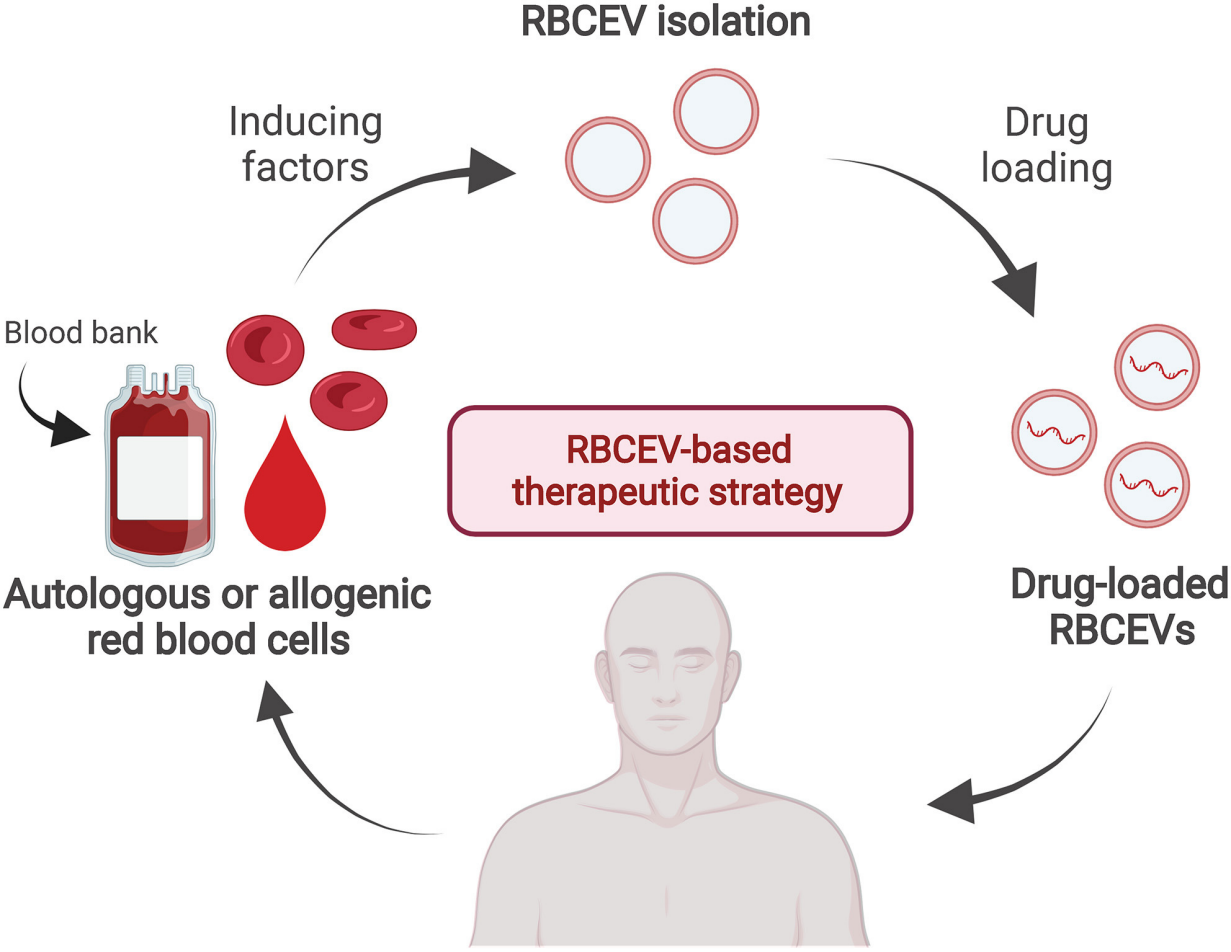
A proposed strategy of drug-loaded RBCEV therapy. RBCs can be collected from a single patient in order to produce autologous RBCEVs and administration back after drug loading to the same patient when required. Alternatively, RBCEVs can be produced in a large scale from the blood group-matched packed red cell units released from the blood bank for the allogenic RBCEV therapy.

In addition to drug delivery, RBCEVs may have other clinical uses, such as biomarkers for diagnosis. Human RBCEVs carrying α-synuclein isolated from patients with Parkinson's disease can cross the blood–brain barrier and impair glutamate uptake *via* an interaction between excitatory amino acid transporter 2 and oligomeric α-synuclein at astrocytic endfeet, leading to reduced synaptophysin levels in the striatum in a mouse model (158). In addition, increased numbers of RBCEVs in blood circulation can indicate hemolytic disorders such as autoimmune hemolytic anemia, complement-mediated hemolysis, malaria, and hereditary erythrocyte membrane disorders, whereas reduced counts were observed in Scott syndrome (cellular calcium abnormality) (21, 159–162). RBCEVs containing miRNA are potential biomarkers for several specific diseases such as cancers, malaria, sickle cell anemia, multiple sclerosis, and diabetes (46). Moreover, circulating MVs originating from RBCs, leukocytes, platelets, or other organs and tissues can serve as potential biomarkers for diagnosis and therapeutic monitoring during the pathogenesis of cardiometabolic diseases and coronary artery disease (163, 164). Moreover, investigating the therapeutic nature of RBCEVs could support the development of therapies combining the basal effects of RBCEVs with specific drugs/functional molecules of interest. In this direction, the RBCEV-based therapeutic strategy is proposed in Figure 2. RBCEVs can be produced from the self-RBCs (autologous) or the blood group-matched packed red cell units (allogenic) and loaded with therapeutic agents, i.e., small molecular compounds, miRNAs, or DNAs before use. Drug-loaded RBCEVs, with the full compatibility to the patients, are administered to pathological tissues in the targeted organs where the drugs are released from EVs to cure the diseases.

In summary, RBCEVs are derived from RBCs, and they contain small amounts of genetic material and proteins. Because of their small size and absence of horizontal gene transfer, RBCEVs represent a good delivery system for carrying drugs to cellular targets with cost-effectiveness, non-immunogenicity, and high stability and biocompatibility. Furthermore, RBCEVs can be easily targeted to every cell type, and they have a short lifespan in the body. Thus, they could be outstanding carriers for drug delivery systems in the future.

## Author Contributions

SC: conceptualization. WC and PN: writing—original draft preparation. SH and SC: writing—review and editing and supervision. WC and SC: visualization and funding acquisition. All authors contributed to the article and approved the submitted version.

## Funding

This study was partially funded by the Office of Higher Education, Science, Research and Innovation Policy Council, Thailand (Grant Number B05F630082 to SC) and the Research Grant for New Researcher, Mahidol University, Thailand (Grant Number A11/2564 to WC). This study was supported by the Faculty of Medicine Ramathibodi Hospital, Mahidol University, Thailand. SC was also financially supported by the Faculty Staff Development Program of Faculty of Medicine Ramathibodi Hospital, Mahidol University, for his research activities. The funders had no role in the design of the study; in the collection, analyses, or interpretation of data; in the writing of the manuscript, or in the decision to publish the results.

## Conflict of Interest

The authors declare that the research was conducted in the absence of any commercial or financial relationships that could be construed as a potential conflict of interest.

## Publisher's Note

All claims expressed in this article are solely those of the authors and do not necessarily represent those of their affiliated organizations, or those of the publisher, the editors and the reviewers. Any product that may be evaluated in this article, or claim that may be made by its manufacturer, is not guaranteed or endorsed by the publisher.
